# Genetic mechanisms underlying increased microalgal thermotolerance, maximal growth rate, and yield on light following adaptive laboratory evolution

**DOI:** 10.1186/s12915-022-01431-y

**Published:** 2022-10-28

**Authors:** Robin Barten, Dirk-Jan M. van Workum, Emma de Bakker, Judith Risse, Michelle Kleisman, Sofia Navalho, Sandra Smit, Rene H. Wijffels, Harm Nijveen, Maria J. Barbosa

**Affiliations:** 1 Bioprocess Engineering & AlgaePARC, Wageningen University and Research, PO Box 16, Wageningen, 6700 AA The Netherlands; 2 Bioinformatics Group, Wageningen University and Research, PO Box 633, Wageningen, 6700 AP The Netherlands; 3 Biosciences and Aquaculture, Nord University, N-8049 Bodø, Norway

**Keywords:** Adaptive laboratory evolution, Microalgae, Temperature, *Picochlorum*, Photobioreactor, Genome assembly, Variant calling, Transcriptomics, Copy number variation

## Abstract

**Background:**

Adaptive laboratory evolution (ALE) is a powerful method for strain optimization towards abiotic stress factors and for identifying adaptation mechanisms. In this study, the green microalga *Picochlorum* sp. *BPE23* was cultured under supra-optimal temperature to force genetic adaptation. The robustness and adaptive capacity of *Picochlorum* strains turned them into an emerging model for evolutionary studies on abiotic stressors such as temperature, salinity, and light.

**Results:**

Mutant strains showed an expanded maximal growth temperature of 44.6 °C, whereas the maximal growth temperature of the wild-type strain was 42 °C. Moreover, at the optimal growth temperature of 38 °C, the biomass yield on light was 22.3% higher, and the maximal growth rate was 70.5% higher than the wild type. Genome sequencing and transcriptome analysis were performed to elucidate the mechanisms behind the improved phenotype. A de novo assembled phased reference genome allowed the identification of 21 genic mutations involved in various processes. Moreover, approximately half of the genome contigs were found to be duplicated or even triplicated in all mutants, suggesting a causal role in adaptation.

**Conclusions:**

The developed tools and mutant strains provide a strong framework from whereupon *Picochlorum* sp. *BPE23* can be further developed. Moreover, the extensive strain characterization provides evidence of how microalgae evolve to supra-optimal temperature and to photobioreactor growth conditions. With this study, microalgal evolutionary mechanisms were identified by combining ALE with genome sequencing.

**Supplementary Information:**

The online version contains supplementary material available at 10.1186/s12915-022-01431-y.

## Background

Microalgal biomass can be processed into renewable lipids, proteins, carbohydrates, and specialty chemicals [1]. Such novel and renewable products become increasingly relevant in the battle against global warming. Despite the opportunities, microalgal production for the bulk product market is not yet competitive due to high operational costs [2, 3]. One major cost factor for production is the need to cool the photobioreactor, due to high levels of solar irradiance. Strains with the capacity to cope with temperatures as found in photobioreactors placed in high-light regions can lead to a significant reduction in operational costs as less cooling is required [3, 4].

Adaptive laboratory evolution (ALE) has been proposed as a high potential tool for improving robustness against a wide variety of abiotic stress factors, such as high temperature [5]. ALE was adopted just recently in microalgal biotechnology but has been proposed as a powerful way to produce genetic mutations that lead to increased fitness in stressful environments [6, 7]. ALE studies commonly yield a rapid shift in the optimal and upper growth temperature of 2–3 °C, whereafter the rate of adaptation reduces significantly [8–11]. The initial rapid adaptation was hypothesized to be caused by pleiotropy [11]. Temperature as a stress factor impacts nearly every cellular process as it affects the protein configuration and membrane fluidity [12]. In addition to the general cellular metabolism, microalgae grow autotrophically through photosynthesis which is considered a thermosensitive process [4]. In prior studies, *Picochlorum* sp. *BPE23* was found to rapidly regulate its photosystems and the formation of storage compounds in order to align the photosynthetic energy production with the energy output to prevent the formation of harmful reactive oxygen species [13].

Significant steps in temperature tolerance are commonly generated by an accumulation of mutations in multiple genes [14]. Next to single nucleotide polymorphisms (SNPs) and genetic variants, copy number variations (CNVs) that are caused by partial genome duplications are hypothesized to drive evolution [15]. The extent to which CNVs contribute to the evolution and phenotypic variation is a topic of scientific interest [16]. In *Arabidopsis thaliana*, CNVs were found to cause significant genome diversity and physiologic differences, which consecutively drove adaptation towards an applied stress factor [15]. CNVs were also proposed to drive adaptation to wastewater in *Chlorella* [17].

In this study, we applied ALE to expand the upper-temperature boundary of the already thermotolerant microalga *Picochlorum* sp. *BPE23* [18]. *Picochlorum* has become one of the most relevant and promising microalgal species over the past years due to its robustness to environmental factors such as high temperature, salinity, and irradiance levels, in combination with its high growth rate. The relevance is also illustrated by the fact that the genome of *Picochlorum* sp. *SENEW3*, a closely related species, became the genome of the month in Trends in Genetics [19]. The species of *Picochlorum* have been used before in evolutionary studies on thermotolerance, halotolerance, and irradiance tolerance due to their robustness and strong adaptation capacity in combination with a small genome size of 13–14 Mb [20–23]. In addition to the phenotypic characterization of growth and biomass composition, we present valuable genomic resources which can be used in future studies. Genome sequencing of both the wild-type and temperature-adapted mutant strains through PacBio HiFi sequencing was performed to generate a phased genome assembly. This assembly was then used to identify genomic mutations and for the subsequent transcriptome analysis. After analysis of all data, hypotheses were formed on the underlying mechanisms behind the improved phenotype of the mutants. This study provides an example of ALE applied to microalgae combined with extensive genetic characterization of mutations to identify potential evolutionary adaptation mechanisms for thermotolerance and domestication.

## Results

### Adaptive laboratory evolution yields an improved mutant strain

ALE was performed for 322 days, during which 293 generations were cultivated (Additional file 1: Fig. S1). The temperature was increased stepwise in small increments after the cell culture fitness recovered from the previous temperature step. In this way, the maximal temperature tolerated by the cell culture shifted from 42 to 44.6 °C at the end of the ALE trajectory.

**Fig. 1:**
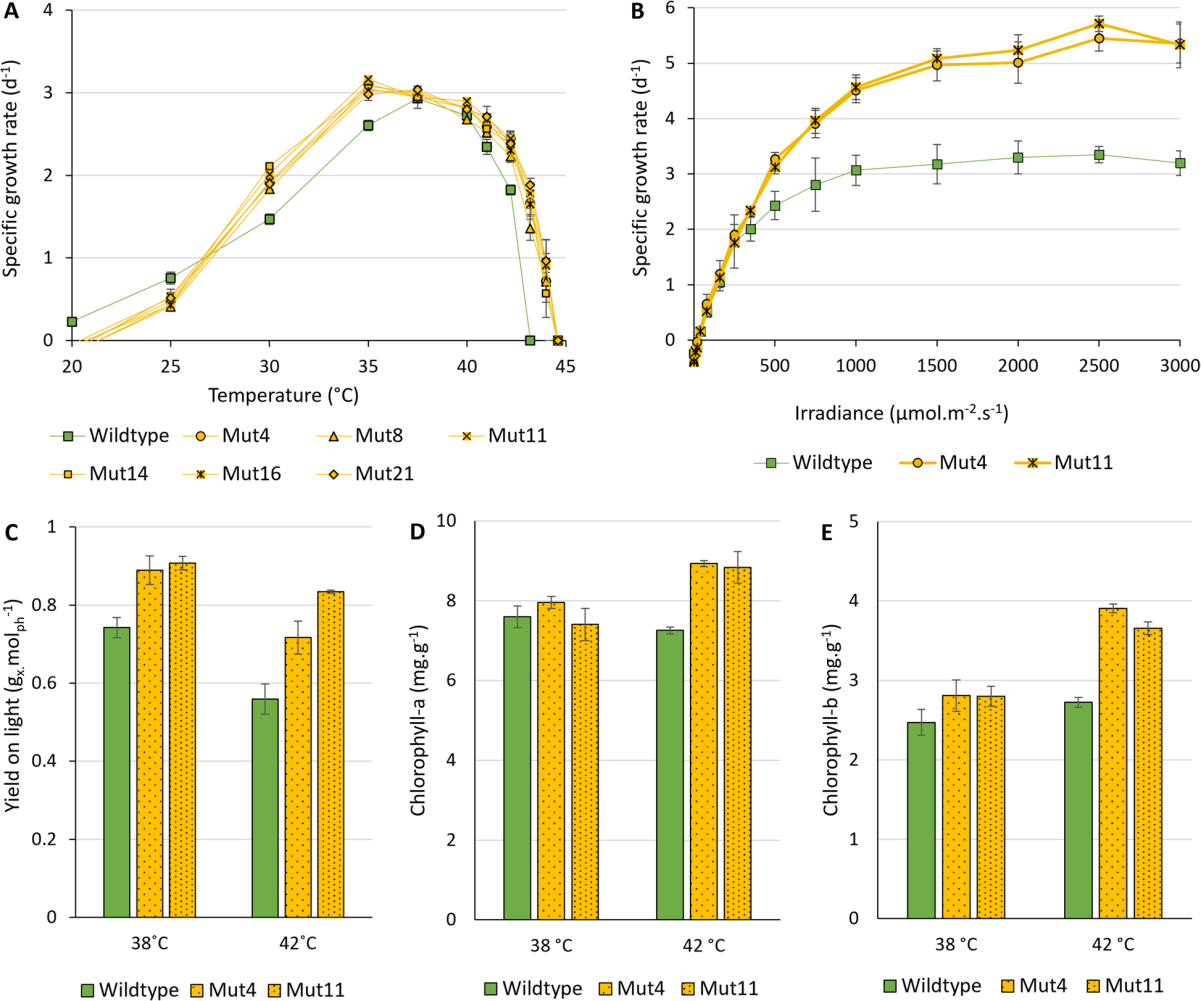
**A** The growth rate was measured for the wild-type and six mutant strains over a temperature range of 20 ﻿°C to 44.6 °C. **B** Photosynthetic irradiance response curves at 38 °C displaying the estimated specific growth rate, as measured by photosynthetic oxygen evolution. **C** The biomass yield on light for growth at 38 °C and 42 °C, the optimal and maximal growth temperature of the wild type, respectively. **D** Chlorophyll-a content in the microalgal biomass. **E** Chlorophyll-b content in the microalgal biomass. The data points represent the average value ± the standard deviation of three biological replicates

After mutant strain isolation, six mutant strains and the wild-type strain were subjected to growth characterization over a temperature range of 20 to 44.6 °C (Fig. 1A). During these screening experiments, a continuous irradiance of 200 μmol m^−2^ s^−1^ was applied. The mutant strains showed an expanded upper-temperature boundary which was expected based on the growth behavior of the cell culture during the ALE trajectory. ALE-induced shifts in the upper-temperature boundary are commonly accompanied by a trade-off at the lower-temperature boundary [24]. Such a trade-off was observed for the mutant strains as they were not able to grow at 20 °C and, in addition, showed reduced growth at 25 °C compared to the wild-type strain. Interestingly, the growth rate at 30 °C and 35 °C was higher for the mutant strains than for the wild-type strain. This increase in growth rate was not expected for these temperatures as selective pressure during ALE was at a supra-optimal temperature. Nonetheless, a broader temperature optimum is advantageous during application for commercial production in photobioreactors due to the fluctuating temperatures in such systems when not controlled. The applied light intensity of 200 μmol m^−2^ s^−1^ is considered a low to average intensity for microalgal cultivation since solar light intensities can fluctuate up to values larger than 2000 μmol m^−2^ s^−1^. Photosynthetic irradiance curves were made under the optimal growth temperature (38 °C) to compare the maximal specific growth rates of the mutant and wild-type strains (Fig. 1B). Mut4 and mut11 displayed a maximal specific growth rate of 5.45 ± 0.23 days^−1^ and 5.71 ± 0.14 days^−1^, respectively, which is significantly higher than the maximal specific growth rate of the wild type, 3.35 ± 0.15 day^−1^. The growth rate of the wild-type strain, grown on nitrate, is comparable to values reported in previous research [25].

Mut4, mut11, and the wild type were further characterized for their growth rate and biomass yield on light in controlled photobioreactors. Biomass yield on light measured at steady state is displayed in Fig. 1C, whereas growth and adaptation trends prior to steady state are presented in Additional file 1: Fig. S2. The growth rate of the wild-type and mutant strains decreased after the temperature change from 38 to 42 °C, as expected based on the growth data displayed in Fig. 1A. Interestingly, the mutant strains reached a new steady state within days without a strong growth rate decrease or a decreased quantum yield, whereas the wild-type strain required 19 days to reach a new steady state with a significant temporary drop in growth and a decreased quantum yield. In fact, two steady-state situations were recognized for the wild-type strain, being at 9 and 19 days after the increase in temperature, with biomass yield on light values of 0.31 ± 0.11 g_*x*_ mol^−1^ and 0.56 ± 0.04 g_*x*_ mol^−1^, respectively (Additional file 1: Fig. S2). In prior research, transcriptomic and compositional analysis after a temperature increase to 42 °C revealed an immediate system-wide stress response, followed by gradual acclimation [12]. A similar response is hypothesized to have taken place in this study, whereby the first steady-state situation at day 9 is considered to be part of the acclimation phase towards the actual steady-state phase at day 19.

The concentration (in mg g^−1^) of both chlorophyll-a and chlorophyll-b, but also all xanthophyll cycle pigments and β-carotene, did not significantly differ between wild-type and mutant strains at 38 °C, despite the higher biomass yield on light (Fig. 1D, E, Additional file 1: Fig. S3). Surprisingly, both mutant strains increased their chlorophyll-a and chlorophyll-b content at 42 °C, indicating that the mutant cells actively increased their photosynthetic light absorbance in response to the temperature increase. Supra-optimal temperature commonly causes photoinhibition to prevent cellular damage through reactive oxygen species (ROS) [26]. This would lead to a severe stress response with decreased growth and reduced photosystems as a result [13]. However, the opposite was observed. Hypothesizing the cellular machinery which copes with excessive heat may have mutated to be more active under this condition, thereby allowing more ROS formation without direct cellular damage. Such waste of light comes at the cost of a reduced photosynthetic biomass yield on light. Microalgal strains naturally exhibit different biomass yields on light [25]. Potential causes for the increased photosynthetic efficiency are increased photochemical efficiency, altered antenna size and filtering pigment composition, and altered biomass composition [4, 27].

Based on growth data and literature examples, potential mechanisms behind the expanded temperature tolerance, the increased maximal specific growth rate, and the increased biomass yield on light are difficult to designate. To elucidate the evolutionary mechanisms underlying the observed physiologic changes, biomass samples were taken during the aforementioned steady states to analyze the mRNA expression levels, gDNA variations, pigment composition, and fatty acid composition).

**Fig. 2:**
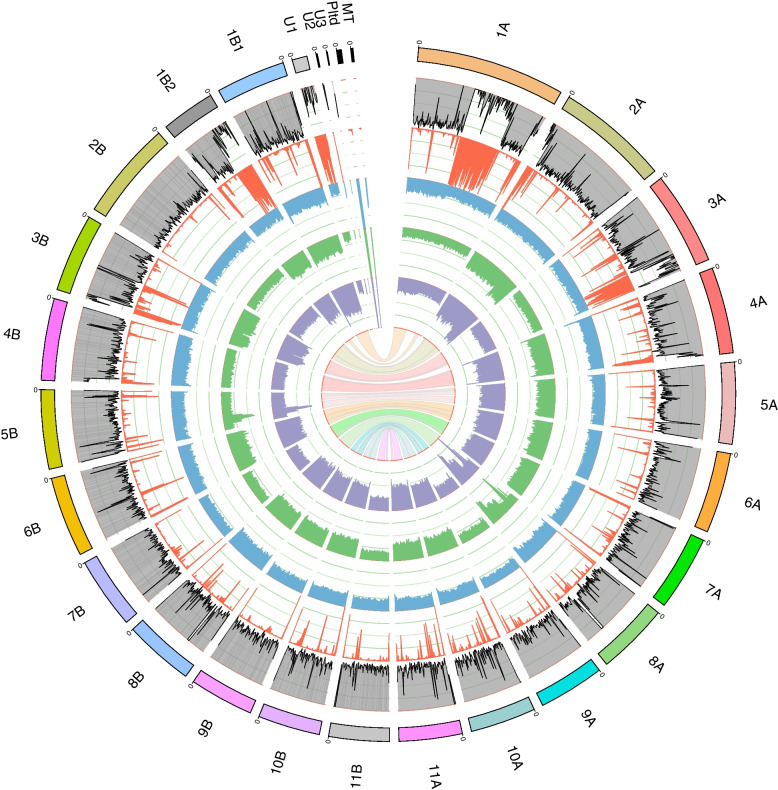
Circos plot to illustrate the phased genome for the wild-type strain and two mutant strains of *Picochlorum* sp. *BPE23*. Items displayed from the outer to the inner layer are (1) assembled contigs, (2) genes density, (3) repeat density, (4) coverage of wild-type sequencing reads, (5) coverage of mut4 sequencing reads, (6) coverage of mut11 sequencing reads, and (7) contig synteny map. Syntenic contigs are displayed with A and B. Contig coding: MT, mitochondrial DNA; Pltd, plastid; U1, U2, U3, unassigned repeat areas

### Partial genome multiplication and gene variants were identified

Using the 199× coverage PacBio HiFi sequencing data with an average length of 18 kb, we produced a fully phased diploid genome assembly for wild-type *Picochlorum* sp. *BPE23*. The assembled contigs, with a total length of 28,372,001 bp, are displayed in Fig. 2. For contig 1A, the long sequencing reads bridged a repeat area, whereas contig 1B1 and 1B2 were not connected. Contig U1, U2, and U3 contain unassigned repeat areas. The plastid and mitochondrial genomes were both assembled in one contig. In the assembly, 14,247 genes were identified that code for 14,443 predicted transcripts, of which 12,283 were annotated with at least one functional protein domain. This is the second phased genome for a species of *Picochlorum* [28].

In addition, mut4 and mut11 were sequenced (PacBio HiFi) with a coverage of 231× and 315× and an average read length of 17 kb, to characterize the genomic differences with the wild type. Interestingly, both mutant strains show a double or even higher coverage of the baseline signal for specific genomic regions, indicating a multiplication of at least 2-fold for nearly half the genome (Fig. 2, tracks 5 and 6). The sequencing coverage of the wild-type strain was constant over nearly the complete genome, indicating accurate assembly and no polyploidy. Both mutant strains exhibited identical genomic multiplication patterns. The genomes of mut8 and mut16 were sequenced using Illumina PE150 and showed the same genome multiplication patterns (Additional file 1: Fig. S5). Microscopic karyotyping is suggested as a follow-up method to study how the partial genome multiplication materialized. Hypothetically, whole chromosomes or chromosome arms were duplicated.

While multiplication of genes with particular functions might be expected in the mutant strains, GO term enrichment analysis did not reveal any significant gene functions. Even the seemingly narrow peaks on contig 5B and 8A with 3 to 4 times genetic multiplication contain genes with a large variety in gene functionality. CNVs are hypothesized to drive adaptation and evolution, as shown in *Arabidopsis thaliana* [15]. Multiple gene copies give mutational freedom as a defective mutation does not immediately result in decreased fitness or cell death, allowing for the accumulation of mutations. In a thermotolerance ALE study with *Saccharomyces cerevisiae*, large genetic duplications were observed in 5 out of 7 mutant strains [8]. However, these genetic duplications were said to have little effect on thermotolerance, and the thermotolerant phenotype was dedicated largely to gene mutations. There is a consensus that after significant genome multiplication, a large number of the non-beneficial duplicated gene copies are lost over time [29]. In a different study with *S. cerevisiae*, such chromosomal duplications disappeared over time as they were unstable and imposed an energetic burden on the cell [30]. The authors speculate that large genetic duplications are acquired as a crude solution to stress, after which more subtle and efficient mutations are accumulated over time that make the chromosomal duplication obsolete.

As all mutant strains show similar growth and thermotolerance patterns, we hypothesize that the improved phenotype originated from mutations that both strains have in common. An overview of in-gene SNPs and nucleotide insertions and deletions is presented in Table 1. In total, 220 mutations were identified of which 154 were intergenic mutations shared by both mutant strains and 45 were found in only one mutant strain. In addition, 15 gene mutations were shared by both mutant strains, and 6 were found in only 1 mutant. Of all mutations, 76.8% are shared between mutant strains, whereas 71.4% of the genic mutations are shared.

**Table 1:**
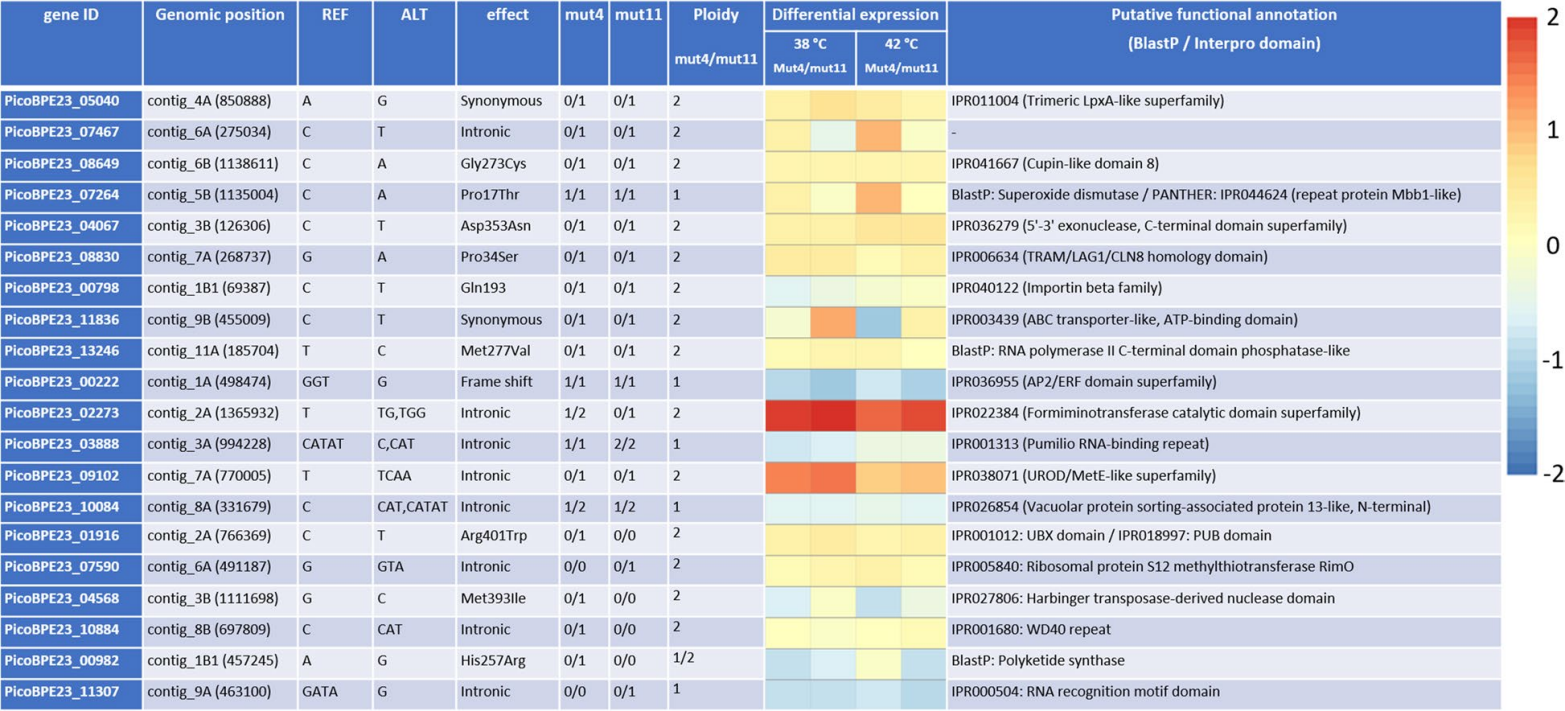
An overview of all mutations that are identified within a gene. Mutations shared by both mutants are listed first, whereas unique mutations follow. The REF and ALT columns display the nucleotide substitutions from reference to alternative. The columns mut4 and mut11 display which REF/ALT variant is present on the haplotypes of the genome where 0 represents the wild-type variant and 1/2 represents the alternative variants. An mRNA differential expression heatmap is displayed showing log2 fold changes of the mutated genes. Lastly, a putative functional gene annotation is presented. Priority was given to BlastP matches, and when no good matches were found, InterPro homology domain annotation was used to infer a gene function

By deducing from the fact that most shared mutations are present on only one of the multiplicated gene copies, it can be concluded that the partial genomic replication took place close to the beginning of the ALE trajectory. Moreover, it is likely that the introduction of the shared mutations between the two mutant strains dates to the moment before the cell lines separated. The large number of shared mutations compared to the non-shared mutations indicates that the parental strain of mut4 and mut11 was dominant in the ALE cell culture until just before the end of the ALE trajectory.

In ALE strain improvement trajectories, the most significant gain in terms of temperature tolerance is gained at the beginning, after which additional mutations have a diminished effect on fitness improvement due to network complexity [7]. Therefore, it is likely that the shared mutations gained at the start of the ALE experiment caused the thermotolerant phenotype.

Most of the identified mutations were found in intergenic non-coding regions at the ends of a contig while few mutations were found in regions between coding genes. Most intergenic DNA has no currently known function. However, some intergenic DNA stretches are known to enhance the translation of nearby genes [31]. Non-coding RNA was demonstrated to regulate acclimation to heat [32]. There were a few intergenic mutations present close to coding genes. We found 249 non-coding RNAs in the assembled genome of *Picochlorum* sp. *BPE23*, but none of the identified mutations was present in a non-coding RNA region. It is therefore unlikely that mutations in intergenic regions were responsible for the improved phenotype. However, the current state of knowledge on intergenic regions does not allow for finite conclusions about the impact of intergenic mutations.

Of the 21 mutated genes, only few showed significant up- or downregulation with log2 fold change values of 1–2 times (Table 1). Most mutations involve a SNP that introduces an amino acid change in the protein whereas a few mutations involve nucleotide insertions/deletions. Three genic mutations and one intronic mutation are unique to mut4, and two intronic mutations are unique to mut11 (PicoBPE23_07590 and PicoBPE23_11307). Moreover, two intronic mutations occurred at the same location in both mutant strains with different nucleotide substitutions. The differences between mutant strains are of special interest as mut11 showed a higher biomass yield on light at 42 °C and performed slightly better than mut4, in general. Mut4 had extra mutations in introns of genes associated with formiminotransferace catalytic domain superfamily, pumilio RNA-binding repeat, ribosomal protein S12 methylthiotransferase RimO, and RNA recognition motif domain. Lastly, the ploidy of polyketide synthase is 2 for mut11. None of these genes exhibits differential expression.

### mRNA expression levels

**Fig. 3:**
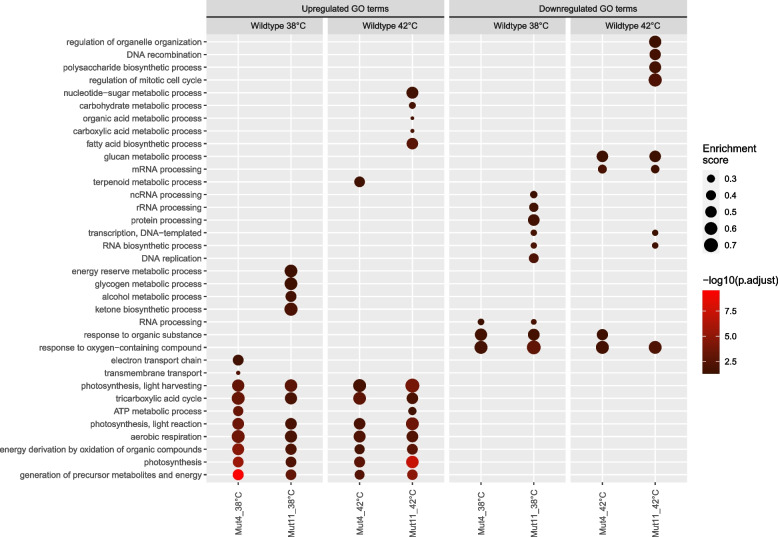
Gene Ontology enrichment analysis displaying up- and downregulated biological processes with the wild-type strain as the base expression and the mutant strains as the variable. Double and irrelevant GO terms were removed from the analysis. The dot size indicates the enrichment score of the GO term whereas the color brightness indicates significance (-log10(FDR))

Principal component analysis confirmed that the mRNA expression patterns of the two mutant strains are very similar to each other, both at 38 °C and 42 °C, but clearly distinct from the mRNA expression patterns of the wild-type strain (Additional file 1: Fig. S6). GO term enrichment was performed to investigate the mRNA expression activity at a process scale (Fig. 3). The mutant strains showed comparable transcription patterns, with minor differences. It must be noted that most GO terms enriched in only one mutant strain have a *p*-value that is relatively close to the cutoff value.

Both wild-type and mutant strains suppressed the processes associated to photosynthesis and the central metabolism in response to a supra-optimal temperature of 42 °C. In addition, many RNA processing and protein processing processes were activated. Regardless of the expanded maximal growth temperature of the mutant strains, 42 °C is still a supra-optimal temperature for both the wild-type and the mutant strains by which reduced metabolic activity was expected. While photosynthesis and metabolism were downregulated in both the wild-type and the mutant strains in response to increased temperature, the wild-type strain showed stronger downregulation.

When comparing the mutant strains to the wild-type strain, many biological processes associated to photosynthesis and energy metabolism were upregulated in the mutant strains, both at 38 °C and at 42 °C (Fig. 3). The increased transcriptional activity of photosynthesis and the energy metabolism corresponds to the observation that mutant cells grew faster with a higher yield on light. While not significantly upregulated as a pathway, several genes associated with nitrogen assimilation and amino-acid biosynthesis were upregulated in the mutant strains. On the other hand, the downregulation of response to oxygen-containing compounds in both mutant strains indicates a lower necessity for ROS deactivation mechanisms. Both mutant strains have downregulated mRNA processing activity. Moreover, mut11 displays downregulation of a wide variety of RNA processing-associated biological processes. Among the downregulated RNA-associated biological processes was non-coding RNA, which is assumed to be involved in regulating gene activity in response to abiotic stress such as heat [33]. Moreover, mut11 shows downregulation of protein processing processes, associated to the proteasome complex, peptidase complex, and endopeptidase complex. The general perception is that protein degradation is necessary in the wild-type strain to break down damaged proteins as a result of heat stress [4, 32].

Interestingly, we did not observe significant overexpression in genes associated with a heat-shock response. In previous research, we exposed the wild-type strain of *Picochlorum* sp. *BPE23* to 5 days of 42 °C and monitored the cell physiology whereby we observed a severe system-wide heat-shock response on a transcription, growth, and cell composition level [12]. The absence of a heat-stress response in this study confirms that our approach to wait for a steady state (19 days) before sampling was required to be able to compare the growth of the mutant strain to the wild-type strains at an optimal and supra-optimal temperature without disturbance of a heat-shock response.

## Discussion

### Adapted mechanisms underlying the physiology improvement

Both the CNVs and the gene mutations can theoretically have caused the thermotolerant phenotype. As several hard selection events (i.e., temperature increases) were applied throughout the ALE trajectory, it is hypothesized that not one but multiple consecutive adaptation events are responsible for the final improved phenotype. Increased selective pressure causes a wipe-out event after which part of the cell lines in the culture are eliminated [34]. Based on cell function, a selection of mutated genes is hypothesized to impact microalgal thermotolerance and growth in general. Due to the diploid nature of *Picochlorum* sp. *BPE23* deleterious mutations can be compensated for by the second copy of the gene on the second allele. This effect induces uncertainty in the impact of the observed mutations. Moreover, microalgal species do not have the same annotation quality as industrialized model organisms such as *Escherichia coli* and *S. cerevisiae*. Nonetheless, strong predictions on gene function can be made based on protein homology and protein domains.

### Adaptation to temperature stress

The effect of heat on cells is system-wide but impacts some cellular components and processes more severely, whereby cells have evolved adaptation mechanisms [12]. The cell membranes are among these components as temperature affects membrane fluidity and therefore unbalances many biological processes. In response, cells remodel the cell membrane composition. Three gene mutations were associated with temperature tolerance specifically. We identified a SNP in PicoBPE23_07264 (ceramide synthase), inducing a Pro34Ser substitution. This gene encodes for the waxy sphingolipid ceramide. In *S. cerevisiae*, de novo synthesis of ceramide synthase was shown to be elevated after heat stress [35].

Due to heat-induced unbalancing in the photosynthetic machinery and the electron transport chain, ROS are formed causing oxidation of cellular components [4]. Photosynthetic organisms downregulate their photosystems to prevent this negative impact which naturally leads to reduced growth. Superoxide dismutase performs an important stress-relieving function by degrading ROS that form due to heat stress [36]. The Pro17Thr substitution to PicoBPE23_07264 could affect its function or rate. This gene’s expression levels increased slightly in the mutant strains. Also, PicoBPE23_13246, encoding for a putative RNA polymerase II C-terminal domain phosphatase-like 4, was found mutated. While intuitively one does not associate this enzyme to heat tolerance, several studies in *A. thaliana* mutator strains showcased its role in tolerance to ROS-inducing abiotic stress factors such as heat through interaction with specific transcription factors and regulatory proteins [37].

### Regulatory genes

Several mutations were identified in genes associated with mRNA transcription and regulation. The mRNA expression of PicoBPE23_03888, associated with mRNA stability regulation, was halved in the mutant strains indicating a lower requirement of the specific mRNA. The transcriptional activator PicoBPE_00222 was found to have undergone a frameshift mutation in both haplotype versions. A study in *A. thaliana* showed that mutagenesis of this gene caused temperature sensitivity [38]. Arguably, mutations in this gene could contribute to thermotolerance. Transcriptional activators and regulators are often found mutated in thermotolerant ALE-derived mutant strains [8].

### Photosynthesis, carbon fixation, and the energy metabolism

Transcriptome analysis revealed many upregulated biological processes associated with photosynthesis, carbon fixation, and energy metabolism in the mutant strains. These were upregulated both at 38 °C and 42 °C compared to the wild type. While most differentially expressed genes had a log2 fold change below 2, a selection of genes was upregulated more severely. Both haplotype versions of RuBisCO, PicoBPE23_01132, and PicoBPE23_00347 were among the most upregulated transcripts with approximately 8 times higher expression. Transcript expression levels were equal for both genes while only the gene on contig 1B1 had undergone a copy number duplication, indicating that transcription was regulated based on the cellular demand for the enzyme. Comparable observations were made for other carbon fixation-associated genes and photosynthesis, glycolysis, and citric acid cycle-associated genes.

Two genes putatively involved in photosynthesis were mutated: PicoBPE23_09102 and PicoBPE23_07264. The first gene contains a UROD domain which catalyzes a step in chlorophyll synthesis whereas the second gene is putatively involved in the regulation of multiple photosystem II subunits, as shown by homology to *Chlamydomonas* [39]. However, both genes show even stronger sequence similarity to other genes, showcasing the difficulty of interpreting genetic data for non-model organisms. None of the gene mutations points exclusively at photosynthesis. While large-scale CNVs do not directly lead to increased enzyme production due to product-regulated mRNA expression, such an event inevitably has a significant effect on cellular physiology. Ultimately, microalgae are capable of regulating energy uptake through antenna size alteration to suit the growth conditions and prevent metabolic imbalance [13, 22]. The observed upregulation of transcription photosynthesis, carbon fixation, and energy metabolism and the increased chlorophyll concentration at 42 °C could also result from pathways downstream or stress-relieving mechanisms that became more efficient under specific growth conditions, thereby allowing all energy uptake mechanisms to be upregulated by the cell.

### Nitrogen metabolism and amino acid biosynthesis

We hypothesize that in addition to evolution to supra-optimal temperature, evolution took place towards growth in the photobioreactor systems and to the microalgal growth medium. The wild-type *Picochlorum* sp. *BPE23* was isolated from Bonairean waters and was not cultured extensively in the laboratory prior to ALE, by which we assume that the wild-type strain did not have mutations that benefit growth on microalgal growth medium. It is known that ALE reproducibly yields side effect mutations that are beneficial for growth in bioreactors on a minimal medium [5]. In the case of *E. coli*, increased substrate utilization efficiency in adaptation to glucose is a recurring observation, among other mechanisms [14]. Light was the energy-providing substrate for our photoautotrophic cultivations, whereas nitrate was added to provide nitrogen for de novo amino acid assimilation. Several mutations can be associated with nitrogen metabolism and amino acid biosynthesis, and several genes associated with these pathways were severely overexpressed. Two mutated genes were involved in amino acid synthesis (PicoBPE23_09102 and PicoBPE23_02273). Moreover, three mutated genes were associated with the transport of cellular compounds and proteins (PicoBPE23_00798, PicoBPE23_11836, and PicoBPE23_10084).

### Confirmation of mutations

Recurring ALE mutations in microalgal strains are not yet known, but their identification becomes increasingly feasible due to the increasing number of ALE studies. Many recent ALE studies have applied genetic sequencing methods such as transcriptomics or genome resequencing to identify mutations [5]. However, these studies were performed with varying microalgal species and ALE targets. With this elaborate case study, we provide strong arguments for evolutionary mechanisms towards both temperature and growth in a minimal medium. Looking forward, more ALE studies with closely related species are required to elucidate recurring adaptation mechanisms to supra-optimal temperature and growth in photobioreactors.

To validate the hypothesized impact of the mutated genes on thermotolerance, they should be genetically engineered in the wild-type strain. Recently, genetic modification tools have been developed for a closely related strain, *Picochlorum renovo* [23]. A strong example of an efficient engineering approach to study the impact of ALE mutations on thermotolerance is demonstrated in *E. coli* by the use of the MAGE technology [14]. After genetic engineering of *E. coli* with the mutations as identified after ALE, an enrichment under selective pressure was followed by resequencing of the cell culture to establish the presence of the mutated genes in the cell culture for their impact on fitness.

## Conclusions

ALE with supra-optimal temperature as a selective pressure was used to create thermotolerant strains of *Picochlorum* sp. *BPE23*. The mutant strains had an expanded maximal growth temperature of 44.6 °C, whereas the maximal growth temperature of the wild type was 42 °C. The mutant strains have a superior maximal growth rate. Moreover, the mutant strains have an increased biomass yield on light at the optimal and maximal growth temperature. The mutant strains showed partial genome multiplication and 21 genic mutations. Based on protein homology, several mutations were associated with mechanisms that could be responsible for increased growth or thermotolerance. Transcriptome analysis showed that photosynthesis and metabolic processes were upregulated in the mutant strains compared to the wild type, while the heat stress-associated processes were downregulated. We hypothesize that evolution towards supra-optimal temperature occurred and was accompanied by evolution towards microalgal growth medium.

From an applied point of view, the improved mutant strains can replace the wild-type strain for biomass production. The biomass yield on light of mut11 increased from 0.74 to 0.91 g_*x*_ mol^−1^ at 38 °C, which directly improves biomass productivity. The broader temperature optimum of the mutant strains stimulates the productivity levels over a wider temperature range, whereby less strict photobioreactor process control is required. Moreover, the maximal specific growth rate of mut11 increased from 3.35 ± 0.15 day^−1^ to 5.71 ± 0.14 day^−1^, allowing for faster upscaling of cell cultures in addition to faster strain improvement trajectories.

With this study, a strong platform was created to enhance the development of *Picochlorum* sp. *BPE23*. A phased genome was assembled to enable future projects using targeted genetic strain modification and fundamental genetic research.

## Methods

### Cell cultivation

#### Growth media and inoculum preparation

*Picochlorum* sp. *BPE23*, isolated from a coastal bay on Bonaire, was pre-cultivated in shake flasks in an orbital shaker incubator (Multitron, Infors HT) under continuous light at an intensity of 100 μmol m^−2^ s^−1^ photosynthetically active radiation (PAR) [18]. The temperature was set at 40 °C, and the relative humidity of the air in the headspace was set to 60% and enriched with 2% CO_2_. Cells were cultured in artificial seawater enriched with nutrients and trace elements. Elements were provided at the following concentrations (in g L^−1^): NaCl, 24.50; MgCl_2_·6H_2_O, 9.80; Na_2_SO_4_, 3.20; NaNO_3_, 2.12; K_2_SO_4_, 0.85; CaCL_2_.2H_2_O, 0.80; KH_2_PO_4_, 0.23; Na_2_EDTA·2H_2_O, 0.11; NaFeEDTA, 3.96·10^−2^; MnCl_2_·2H_2_O, 1.71·10^−3^; ZnSO_4_·7H_2_O, 6.60·10^−4^; Na_2_MoO_4_·2H_2_O, 2.42·10^−4^; Co(NO_3_)_2_·6H_2_O, 7.00·10^−5^; NiSO_4_·6H_2_O, 2.63·10^−5^; CuSO_4_·5H_2_O, 2.40·10^−5^; K_2_CrO_4_, 1.94·10^−5^; Na_3_VO_4_, 1.84·10^−5^; and H_2_SeO_3_, 1.29·10^−5^. HEPES (4.77 g L^−1^) was added to Erlenmeyer cultures as a pH buffer. The medium pH was adjusted to 7.0, after which it was filter sterilized before use. During photobioreactor cultivation, Antifoam B (J.T.Baker, Avantor, USA) was added at a concentration of 0.5 mL L^−1^ from a 1% w/w% stock. Also, 0.168 g L^−1^ Sodium bicarbonate (NaHCO_3_) was added at inoculation to provide sufficient CO_2_ at the start of the cultivation. The photobioreactor was inoculated at a starting cell density of OD_750_ 0.2.

#### Adaptive laboratory evolution and mutant isolation

ALE was performed in heat sterilized flat-panel photobioreactors with a 1.8-L working volume, a 20.7-mm light path, and a 0.08-m^2^ surface area for irradiation (Labfors 5 lx, Infors HT, Switzerland) [13]. The incident irradiance was set at a continuous rate of 813 μmol m^−2^ s^−1^ (PAR). This irradiance rate was chosen as the total quantity of light is then equal to the total light of an average day in the Caribbean but distributed homogeneously over 24 h instead of as a diel pattern. The cell density was kept within a certain range through automatic photobioreactor dilution based on the penetration of light through the cell culture (Turbidostat mode). The photobioreactor was diluted to maintain a light penetration level of 10 μmol m^−2^ s^−1^ (PAR). The photobioreactor was aerated through sparging of compressed air at a rate of 980 mL min^−1^. CO_2_ was provided on demand through indication of an increased pH. The reactor pH was set at 7. The temperature was increased stepwise throughout the evolution process for 322 consecutive days as follows: 30 °C, 42 °C, 42,5 °C 43 °C, 43.5 °C, 44 °C, 44.2 °C, 44.7 °C, 44.2 °C, 44.4 °C, 44.6 °C, and 44.8 °C (Additional file 1: Fig. S1). At 44.6 °C, a sample was taken from the photobioreactor, and cells were plated on agar plates followed by incubation at 42 °C for 1 week. Colonies were picked, transferred into liquid growth medium, and subjected to a growth screening using the HT24 incubating unit (Algenuity, UK) in which a screening temperature of 42 °C and a light intensity of 200 μmol m^−2^ s^−1^ (PAR) were applied. The 6 strains with the highest growth rate were selected for more elaborated growth rate and thermotolerance characterization.

#### Growth rate screening—Erlenmeyer cultivation

The wild-type and six mutant strains with numbers 4, 8, 11, 14, 16, and 21 were cultured at 20 °C, 25 °C, 30 °C, 35 °C, 37.5 °C, 40 °C, 41 °C, 42.2 °C, 43.2 °C, 44 °C, and 44.6 °C to determine the maximum growth rate over a temperature range. Cells were cultured in 250 mL Erlenmeyer flasks with a liquid volume of 100 mL. Repeated batch cultivation was applied in which cultures were diluted daily to a cell concentration of OD_750_ 0.05. The OD_750_ was measured at 6 and 24 h after inoculation. Broad-spectrum white fluorescent light (Philips master TL-D reflex, color temperature 840) was set at a continuous intensity of 200 μmol m^−2^ s^−1^ (PAR). A 2% CO_2_ headspace was created. Data acquisition was made at steady-state [18].

#### Characterization of mutant strains—photobioreactor operation

Sterilized flat panel photobioreactors (Algaemist; Technical Development Studio, WUR) with a 0.38-L working volume and a 14-mm optical depth were used for characterization and comparison of growth characteristics and RNA expression patterns of the wild-type and two evolved strains of *Picochlorum* sp. *BPE23* [25]. Cells were grown in turbidostat mode with similar growth conditions as in the “Adaptive laboratory evolution and mutant isolation” section, with the exception that air was supplied at a rate of 200 mL min^−1^. Experiments were performed as biological triplicates, and samples were taken in a steady-state for both 38 °C and 42 °C. Temperature was initially set at 38 °C until samples were taken, after which it was increased to 42 °C until a new steady state was reached.

### Offline measurements and analysis

The quantum yield, cell size, cell number, and biomass concentration were measured as described in [13]. Biomass harvest and biomass lyophilization, determination of pigments through HPLC, and determination of fatty acids through GC was performed as described in [12]. The photosynthesis irradiance curve and the maximal specific growth rate were measured as described in [25].

### Genomic DNA analysis

#### Genome sequencing

gDNA extraction and sequencing were performed by the genomics facility of WUR Bioscience (Wageningen, The Netherlands). gDNA of the wild type, mut4, and mut11 was sequenced through PacBio sequencing. Fifty milligrams of washed and lyophilized biomass was used for gDNA extraction using MagMax Plant DNA kit (Applied Biosystems). Extracted gDNA was sheared using a megadisruptor device (Diagenode), aiming for a 25-kb fragment size. Barcoded SMRT bell template libraries were made for each microalgal strain using SMRT bell express template prep kit 2.0 (PacBio). gDNA was eluted in 20 μL elution buffer and pooled for size selection on a Blue Pippin instrument (Saga Sciences). The 8 to 13 kb and ≥ 13 kb fractions were collected for each lane after which purification and concentration were performed with Ampure XP beads (Beckman Coulter). Purified SMRT bells were finally analyzed for size and used for primer annealing, polymerase binding, and purified using AMPure PB beads (SMRTLink10.1). Sequencing was done on one SMRT cell of a PacBio Sequel IIe system with 65 pM on plate loading by diffusion with standard settings and 30-h movie time. Data demultiplexing and HiFi read processing were done by SMRT Link 10.1.

Illumina paired-end sequencing of the wild type, mut4, mut8, mut11, and mut16 with 150 bp reads was performed by Novogene (Novogene, UK). For library preparation, DNA was randomly fragmented to a size of 350 nt, then end polished, A-tailed, ligated with Illumina sequencing adapters, followed by PCR enrichment of P5 and P7 oligos. The PCR product was purified (AMPure XP system) and sequenced.

#### Genome assembly

The genome assembly of wild-type *Picochlorum* sp. *BPE23* was reconstructed by assembling the HiFi reads using Flye 2.9-b1768 with default settings [40]. Next, Illumina sequencing data of the wild-type strain were used to polish the Flye genome assembly with Pilon v1.24 with argument “--fix all” [41]. For polishing, the paired-end Illumina reads were trimmed with Trimmomatic v0.39 “ILLUMINACLIP:TruSeq3-PE.fa:2:30:10 LEADING:3 TRAILING:3 SLIDINGWINDOW:4:15 MINLEN:36” and mapped to the assembly with BWA-MEM2 v2.2.1 [42, 43]. Sorting and indexing of mapping files were done with SAMtools v1.12 [44]. The chloroplast and mitochondrial gDNA were labeled by mapping to the assembly of *Picochlorum* sp. *“soloecismus”* strain DOE 101 with minimap2 “-xasm20” [45]. The identity of the chloroplast and mitochondrial gDNA fragments was then confirmed by a BLASTN 2.11.0+ hit to the NCBI nt database [46]. Homologous contigs were identified by leveraging the location of the duplicated BUSCO genes followed by self-alignment with minimap2 v2.10 (“-k19 -w19 -A1 -B9 -O16,41 -E2,1 -s200 -z200 -X”) [47]. To assess the completeness of the genome assembly, BUSCO v5.2.2 was used with the chlorophyta_odb10 set as well as KAT v2.4.1 [48, 49]. To further assess the structure of the genome assembly, it was aligned to the closely related genomes of *Picochlorum SENEW3*, *Picochlorum oklahomensis*, and *Picochlorum celeri* [20, 28]. In addition, the original HiFi data was mapped back with minimap2 v2.22. The coverage of the HiFi data was calculated using BEDtools v2.30.0 [50]. HiFi and Illumina data were compared for wild type, mut4, and mut11. Further study towards genome ploidy was performed through KMC v3.1.1 and Smudgeplot v0.2.3 [51, 52].

#### Genome annotation

Before annotation of the wild-type genome assembly, repeats were identified in the polished assembly with RepeatModeler v2.0.2 with argument “-LTRStruct” and subsequently masked with RepeatMasker 4.1.2-p1 [53]. The RNA-seq samples of the wild type were mapped to the genome with HISAT2 v2.2.1 to serve as RNA evidence, whereas the proteome of *Chlamydomonas reinhardtii* (GCF_000002595.2) served as protein evidence for gene prediction [54]. Gene prediction was performed with BRAKER v2.1.6 with argument “--etpmode” [55]. Mitochondrial and chloroplast sequences that were included in RNA-seq data mapping were excluded for gene prediction. Functional annotation was done with InterProScan 5.52-86.0 [56]. The included analyses were TIGRFAM, SUPERFAMILY, PANTHER, Gene3D, Coils, Pfam, and MobiDBLite. In addition, InterPro and Gene Ontology (GO) terms were listed. An organism-specific annotation R package was created using the MakeOrgPackage function from the AnnotationForge Package v1.32.0 [57]. Lastly, non-coding RNAs were identified by searching the genome with Cmscan from the Infernal toolkit against the Rfam database [58].

#### Genome analysis

Variant calling to identify mutations in the ALE strains was performed by aligning HiFi reads to the phased genome assembly with Minimap v2.22 with argument “-ax map-hifi --MD --secondary=no.” Variants were called with GATK v4.3.2.0 HaplotypeCaller, combined with CombineGVCFs and genotyped with GenotypeGVCFs [59]. Filtering variants with GATK SelectVariants was done on QUAL < 1000, homozygous REF allele for wild type, and non-homozygous REF allele for either mutant strain. We used SnpEff 5.0 with the novel wild-type genome and annotation to determine the effect of the mutations on the amino acid sequence [60].

Enrichment analyses were performed with genes that had doubled or tripled coverage in mut4 or mut11 compared to the wild type using ClusterProfiler in R v4.0.4 [61]. MCScanX was used to identify syntenic blocks based on an all-vs-all BLASTP search (Wang et al. 2012) [62].

Circos v0.69-8 was used to visually combine all data on the framework of the wild-type genome assembly [63]. Gene and repeat density were calculated in bins of 10 kb.

### mRNA extraction, sequencing, and analysis

Biologically triplicate biomass samples of wild type, mut4, and mut11 steady-state cell cultures at 38 °C and 42 °C (paragraph 2.1.4) were analyzed for mRNA levels. Biomass pre-treatment, mRNA extraction and sequencing, and data quality control were performed as described by Barten et al. [12]. Fragments of the mRNA library with an average size of 300 bp were sequenced using the Illumina Novoseq PE150 platform, yielding paired-end reads of 150 nt (Novogene, UK).

Paired-end reads were mapped to the phased wild-type genome using HISAT2 v 2.2.1. Transcripts were assembled using StringTie v1.3.2d. StringTie’s prepDE Python script was used to extract raw read counts per gene. Raw counts were normalized using DESeq2 v1.30.1. Pairwise differential expression between mutant strains and wild type at different temperatures was calculated using DESeq2, setting alpha to 0.05. Enriched GO terms were detected using ClusterProfiler’s gseGO function and visualized using the Ggplot2 R package.

## Supplementary Information

**Additional file 1: Figure S1.** Photobioreactor dilution rate and temperature during the adaptive laboratory evolution process. **Figure S2.** The photobioreactor dilution rate during characterization of the wildtype, mut4, and mut11. **Figure S3.** The concentration of pigments in the wildtype and mutant strains at 38 °C and 42 °C. **Figure S4.** The concentration of polar fatty acids in the wildtype and mutant strains at 38 °C and 42 °C. **Figure S5.** Coverage of genome sequencing reads for the wildtype, mut4, mut8, mut11, and mut16. **Figure S6.** PCA bi-plot of the sequenced mRNA samples collected from steady-state cultures at 38 °C and 42 °C.

## Data Availability

Wild-type and mutant strains of *Picochlorum* sp. *BPE23* are stored in liquid nitrogen and are available upon reasonable request. A web portal (https://www.bioinformatics.nl/picochlorum_bpe23), including a genome browser, is available for scientists to use in their research [64]. Included items are the genomes of the wild type, mut4, and mut11. Moreover, all mRNA sequencing data and the identified mutations are included. Moreover, genomic sequence read archives and assemblies are available at NCBI under the umbrella project number: PRJNA872549 (https://www.ncbi.nlm.nih.gov/bioproject/PRJNA872549) [65].

## Abbreviations

ALE: Adaptive laboratory evolution
CNV: Copy number variation
PAR: Photosynthetically active radiation
GO: Gene Ontology
ROS: Reactive oxygen species
SNP: Single nucleotide polymorphism
