# Supplementary material for: Genetic mechanisms underlying increased microalgal thermotolerance, maximal growth rate, and yield on light following adaptive laboratory evolution
Source: BMC Biol. 2022 Oct 28;20:242. doi: 10.1186/s12915-022-01431-y (PMC9615354; doi:10.1186/s12915-022-01431-y)

# Additional file 1

Belonging to the publication: Barten et al. Adaptive laboratory evolution increases microalgal thermotolerance, maximal growth rate, and yield on light: pinpointing genetic mechanisms.

## Additional figure S1

**Figure S1: Photobioreactor dilution rate and temperature during the adaptive laboratory evolution process.** The dilution rate is equal to the growth rate due to the turbidostat operation of the photobioreactor. Temperature was increased step-wise, inducing fitness decreased to the microalgal cell culture.


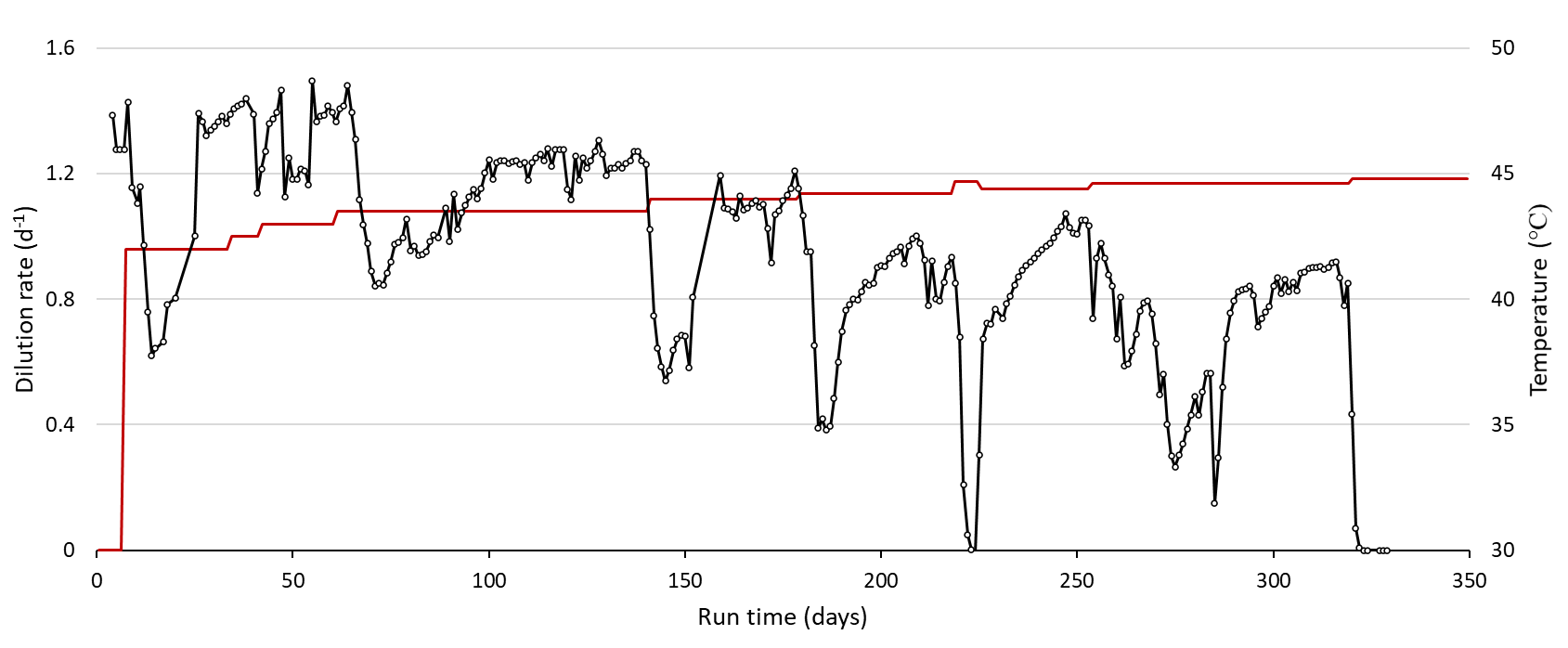


## Additional figure S2

**Figure S2: The photobioreactor dilution rate during characterization of the wildtype, mut4, and mut11.** The dilution rate is equal to the growth rate due to the turbidostat operation of the photobioreactor. The wildtype strain required more time to acclimate to 42 °C


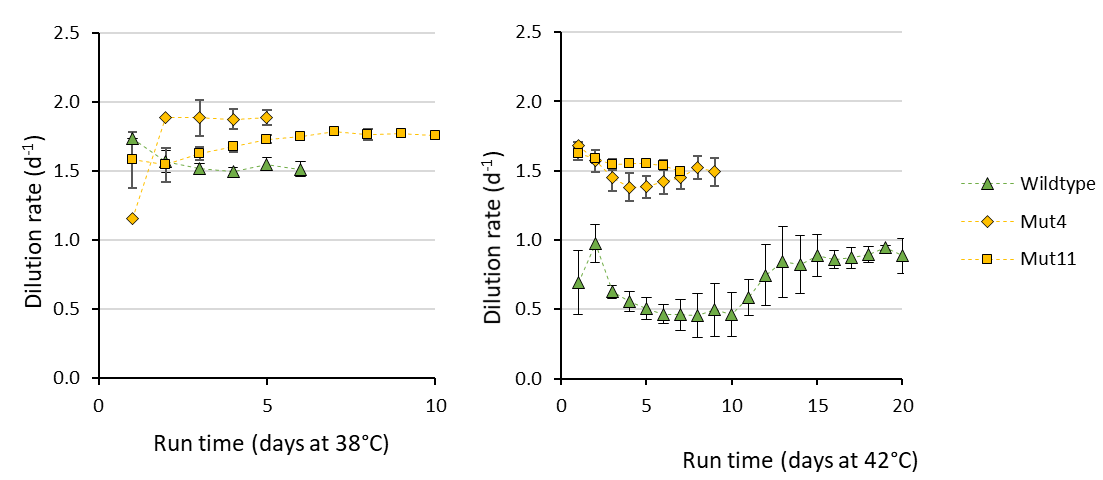


## Additional figure S3

#### Pigmentation

Pigment content plays a large role in the photosynthetic capacity. *Picochlorum sp. BPE23* utilizes both chlorophyll A and B in its photosystems. In addition to chlorophylls, photosystems contain carotenoids. Carotenoids not only fulfill a function in light harvesting but also in protection from cellular damage through quenching of excess energy, filtering of light, and scavenging of reactive oxygen species. Mut4 and mut11 showed an increased chlorophyll-a and chlorophyll-b concentration at 42 °C (Figure S3). The other pigments do not show differences between wildtype and mutants. The chlorophyll concentration falls within normal range compared to other studies on *Picochlorum sp. BPE23* (13). However, the values are on the lower end of the spectrum as chlorophyll A can reach a concentration of up to 20 mg.g^-1^ in both *Picochlorum sp. BPE23* and other microalgal species. Pigmentation is downregulated in the presence of high light. Sampling for pigmentation was done under turbidostat reactor operation. In a turbidostat culture the light per cell is relatively high compared to other modes of operation as light is required at the outgoing end of the photobioreactor for proper dilution of the cell culture.

**Figure S3: The concentration of pigments in the wildtype and mutant strains at 38 °C and 42 °C.** Pigment concentration was measured from biological triplicates during the strain characterization experiment. Values are displayed as the average ± standard deviation.


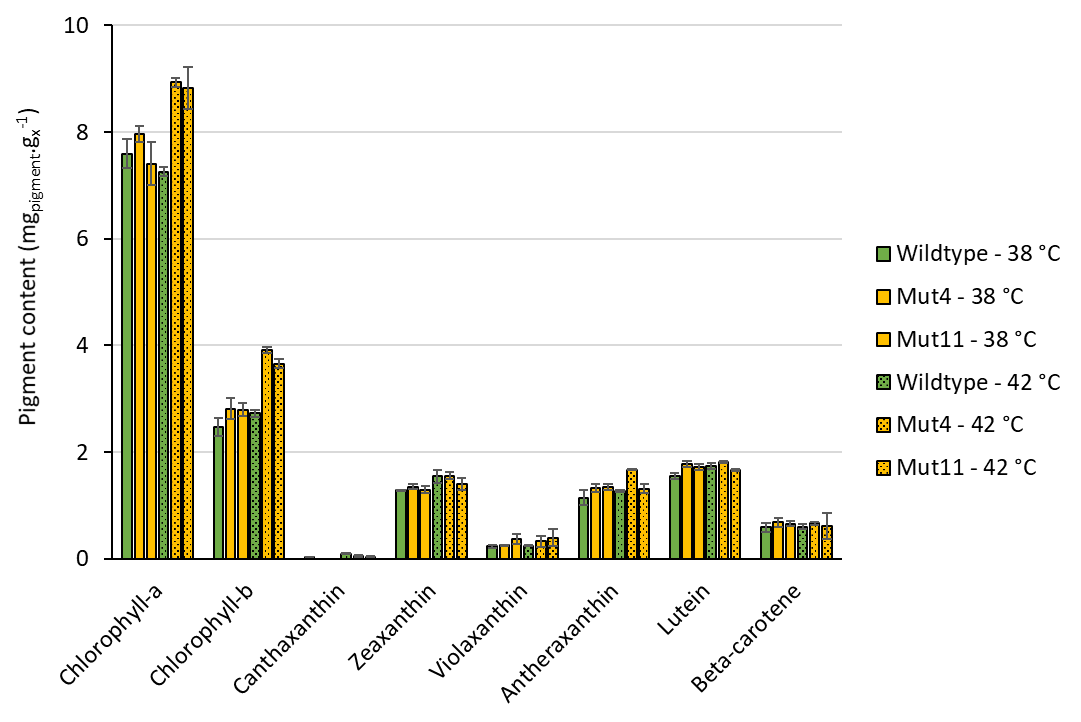


## Additional figure S4

#### Polar fatty acids

Polar fatty acids make up the cell membranes and thylakoid membranes. In previous research the cell membrane composition was found to be altered as a consequence of temperature stress (12, 13). In this study the wildtype strain contained a significantly larger amount of polar fatty acids than the mutant strains at 38 °C with a concentration of 90.4±1.84 mg_lipid_.g_x_^-1^, compared to 65.2±3.13 mg_lipid_.g_x_^-1^ and 67.0±1.21 mg_lipid_.g_x_^-1^ for mut4 and mut11, respectively (Figure S4). At 42 °C the difference became smaller with values of 77.5±3.10, 76.5±0.78, and 69.83±2.85 mg_lipid_.g_x_^-1^, respectively. Especially the polyunsaturated fatty acid C18:3 was present at lower concentrations in the mutant strains at both 38 °C and 42 °C. Reduction of unsaturated fatty acids such as C18:3 is done to decrease the membrane fluidity to counteract the increased fluidity following an increased growth temperature (12, 13).

#### Neutral lipid content

Microalgae store excess energy as neutral lipids in lipid bodies. *Picochlorum sp. BPE23* maintained a very small pool of such lipid bodies under non-stressed growth conditions. The lipid pool fully consists of C16:2 when grown at 38 °C. When the temperature was increased to 42 °C the wildtype strain accumulated 0.8 wt% lipids. Previous research has shown that *Picochlorum sp. BPE23* funnels excessive energy towards fatty acid production under temperature stress to prevent formation of harmful reactive oxygen species. Both mutant strains didn’t accumulate lipids at 42 °C which indicates that there was no cellular stress.


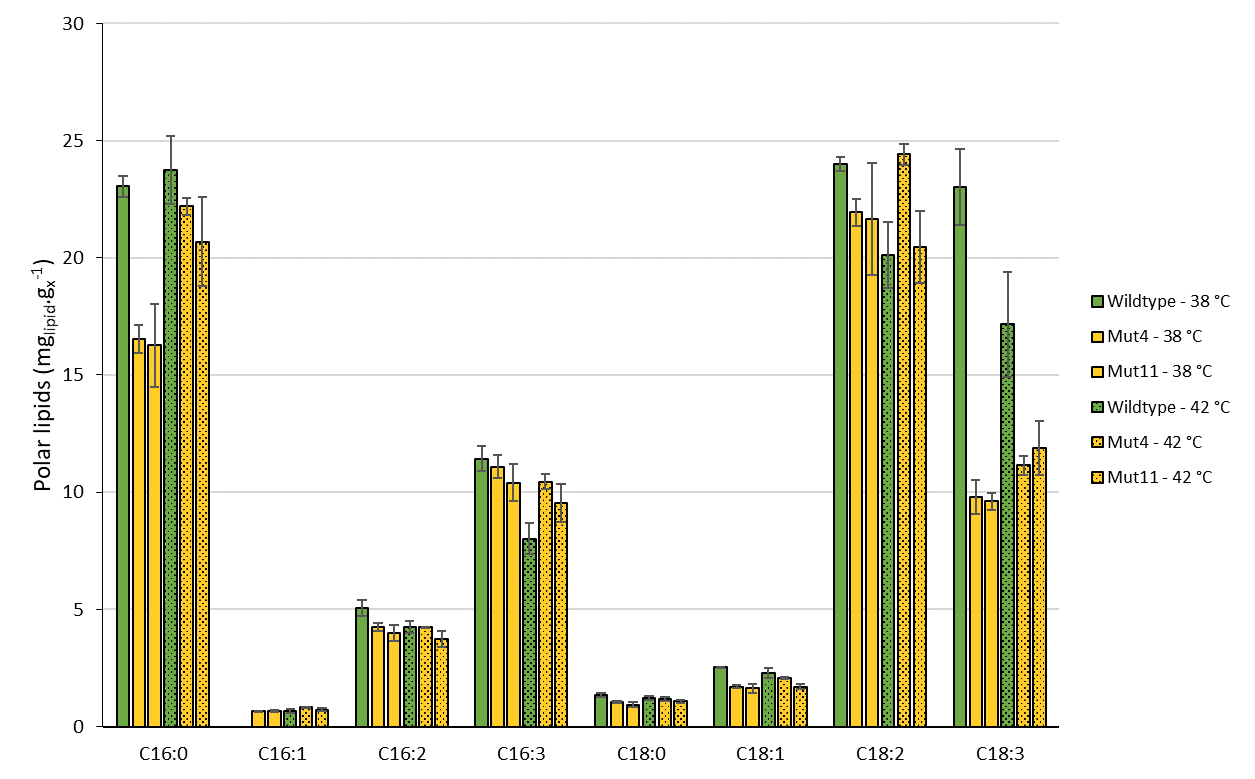


**Figure S4: The concentration of polar fatty acids in the wildtype and mutant strains at 38 °C and 42 °C.** Fatty acid concentration was measured from biological triplicates during the strain characterization experiment. Values are displayed as the average ± standard deviation.

## Additional figure S5

The genomes of four mutant strains and the wildtype strain were sequenced by Illumina PE150 sequencing. The reads were then mapped to the phased diploid reference genome that was created through HiFi sequencing. The sequencing mapping coverage was calculated for each contig and displayed as a heatmap in Figure S5. The mitochondrial and plastid DNA were removed from the heatmap. Interestingly, the sequencing coverage for some of the contigs is exactly double as for others in the mutant strains, whereas the sequencing coverage of the contigs in the wildtype strain falls in a comparable range for each contig. Contigs 6, 8, and 10 had both copies doubled, whereas contig 1, 2, 3, 4, 5, 7, 9, and 11 had one of the contigs doubled. This is consistent with the findings of the HiFi sequencing (Figure 2). Interestingly, the same pattern was observed for each of the mutant strains. Only mutant 8 shows a different pattern with a very low coverage for contig 2B and a very high coverage for contig 2A.


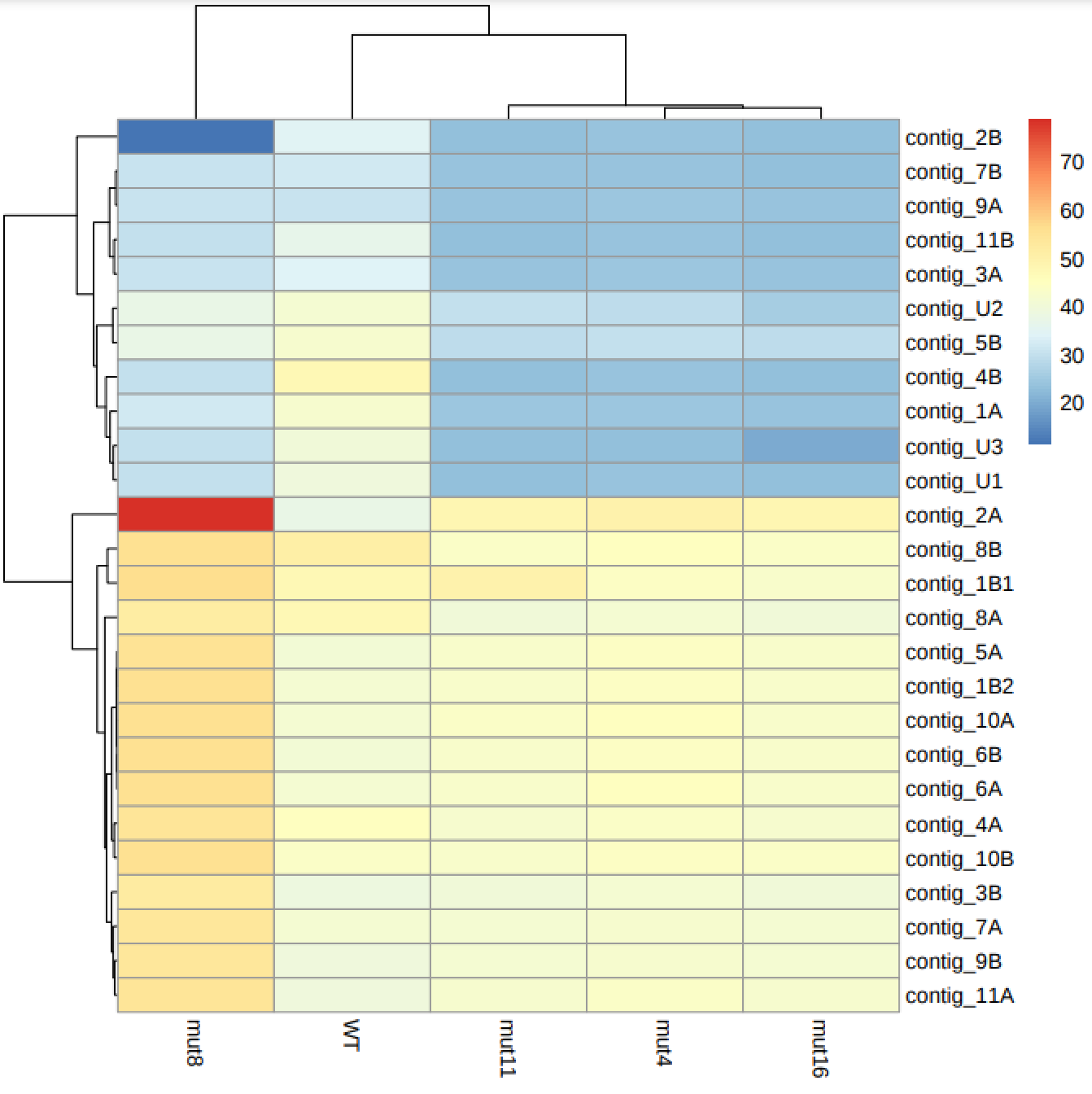


**Figure S5: Coverage of genome sequencing reads for the wildtype, mut4, mut8, mut11, and mut16.** Sequencing was done through Illumina PE150

## Additional figure S6

A principal component analysis was performed using the mRNA sequencing data for quality control and to compare major mRNA expression patterns (Figure S6). All samples clustered together as expected. In addition, mut4 and mut11 cluster together as well indicating comparable mRNA expression patterns. The wildtype samples are different from the mutant strains at both 38 °C and at 42 °C. Two samples were taken at 42 °C as the steady-state situation was not reached before day 19. Interestingly, the sample taken at day 9 for the wildtype is further away from the unstressed situation of 38 °C than the taken at day 19. This indicates that the mRNA expression levels were normalizing and that the hypothesized heat-shock response faded. The direction of the samples in the PCA analysis in response to the temperature increase is similar for both the wildtype and the mutant strains, namely to the top right corner.

#

**Figure S6: PCA bi-plot of the sequenced mRNA samples collected from steady-state cultures at 38 °C and 42 °C.** Both mut4 and mut11 were sampled in a steady-state situation. The wildtype strain was sampled twice at 42 °C, at day 9 and day 19 after the increase in temperature. The first two PCs are displayed.


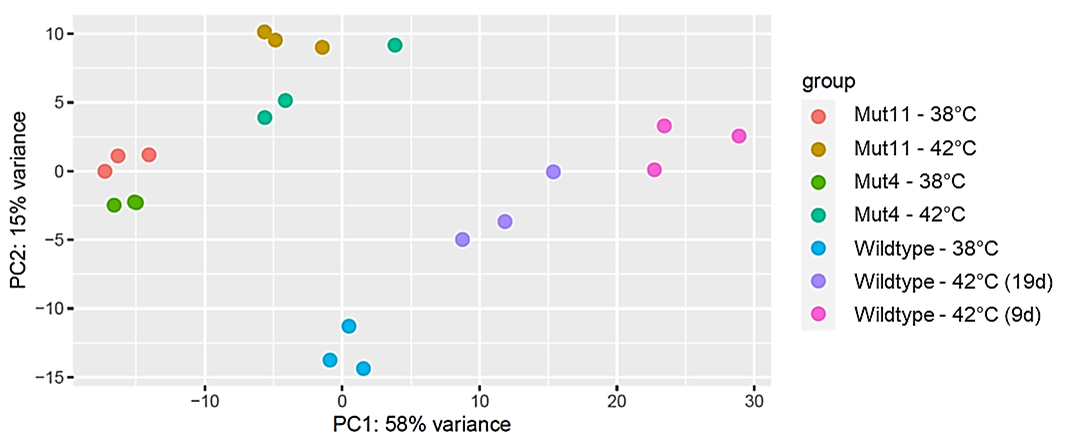

Supplement: Supplementary file 1 — Additional file 1: Figure S1. Photobioreactor dilution rate and temperature during the adaptive laboratory evolution process. Figure S2. The photobioreactor dilution rate during characterization of the wildtype, mut4, and mut11. Figure S3. The concentration of pigments in the wildtype and mutant strains at 38 °C and 42 °C. Figure S4. The concentration of polar fatty acids in the wildtype and mutant strains at 38 °C and 42 °C. Figure S5. Coverage of genome sequencing reads for the wildtype, mut4, mut8, mut11, and mut16. Figure S6. PCA bi-plot of the sequenced mRNA samples collected from steady-state cultures at 38 °C and 42 °C. [file 12915_2022_1431_MOESM1_ESM.docx]
